# International Survey of Eye Care Practitioners on Dry Eye Disease: A Comparative Analysis of South Africa, the United Kingdom and Other Global Regions

**DOI:** 10.3390/jcm15176757

**Published:** 2026-08-31

**Authors:** Christine Croker, Shehzad A. Naroo, Raquel Gil-Cazorla

**Affiliations:** Optometry and Vision Science Research Group, Aston University, Birmingham B4 7ET, UK; 180198379@aston.ac.uk (C.C.); s.a.naroo@aston.ac.uk (S.A.N.)

**Keywords:** diagnosis, management, practitioner confidence, MGD, tear break-up time, ocular surface disease, optometry, cross-sectional study, health disparities

## Abstract

**Background:** Dry eye disease (DED) and meibomian gland dysfunction (MGD) impose a significant global burden, yet clinical practice varies substantially between regions. This study investigated international trends in DED diagnosis and management among eye care practitioners (ECPs), with regional focus on South Africa (SA), the United Kingdom (UK) and the rest of the world (RoW; heterogeneous international respondents). **Methods:** An anonymised cross-sectional online survey was distributed to ECPs globally (May 2023–September 2024). In total, 383 participants from 31 countries were included in the analysis (SA *n* = 137; UK *n* = 112; RoW *n* = 134). Mann–Whitney U tests with Benjamini–Hochberg false discovery rate correction, chi-square tests and Kendall’s tau concordance were applied. **Results:** SA practitioners reported significantly lower confidence across all four DED domains than their UK and RoW counterparts (all *q* < 0.05; Cliff’s delta small-to-medium). Core diagnostic practices—case history (88.7%), fluorescein staining (79.4%) and tear break-up time (77.3%)—were globally consistent. UK practitioners showed greater recognition of blepharitis and higher uptake of lid massage, dietary modification and intense pulsed light. Advanced diagnostics received low reported importance globally. **Conclusions:** Significant regional disparities exist in practitioner confidence, diagnostic emphasis and therapeutic access. A tiered diagnostic framework is proposed to support equitable care, applying a universal core of assessments supplemented by advanced tools where resources permit.

## 1. Introduction

Dry eye disease (DED) is a common multifactorial condition that places a significant economic burden on healthcare systems worldwide [1,2]. Its complex aetiology, coupled with a frequently poor correlation between clinical signs and patient-reported symptoms, makes accurate diagnosis and effective management particularly challenging [3]. Despite advances in research—including the identification of tear biomarkers, the development of targeted therapeutics and the publication of comprehensive global guidelines (TFOS DEWS II [3] and III [4]).

Meibomian gland dysfunction (MGD) is widely recognised as the leading cause of evaporative DED [5]. Prevalence estimates for MGD in populations over 40 range from 38% to 68%, and TFOS DEWS III reports that any MGD (including asymptomatic gland changes) reaches up to 66% in men aged 80 years and older, with significantly higher rates in men than women from age 70 onwards—a male-predominant pattern specific to MGD, as DED overall remains more common in women [6]. Modern lifestyle factors, particularly prolonged digital device use, further amplify the disease burden; a recent review estimated DED prevalence at 26–70% among visual display unit users [7].

Substantial variability in clinical practice persists worldwide [8,9,10,11,12]. Survey-based research has consistently identified fluorescein tear break-up time, ocular surface staining and case history as the core global diagnostic toolkit, while also highlighting persistent underutilisation of advanced tests such as tear osmolarity and MMP-9 [8,9,10,11,12]. Artificial tears remain the global treatment mainstay, yet are frequently insufficient for moderate-to-severe disease, prompting the increasing use of anti-inflammatory agents, lid hygiene regimens and in-office thermal therapies [9,11].

Despite this body of evidence, South Africa (SA) and the wider African continent remain substantially underrepresented in the literature. No published study has directly compared SA and United Kingdom (UK) practitioner perspectives within the same dataset. This gap limits the understanding of local disease burden, diagnostic practices and barriers to equitable care—particularly relevant given SA’s unique environmental profile of moderate-to-high altitude, low humidity and high UV exposure, contrasting with the UK’s cool, temperate climate.

This study aimed to investigate international trends in DED and MGD diagnosis and management, with a regional emphasis on SA and the UK. Specific objectives were to: (i) characterise practitioner confidence across four clinical domains; (ii) compare perceived DED and MGD prevalence and symptom profiles; (iii) examine regional variation in diagnostic test importance and treatment uptake; and (iv) identify factors influencing diagnostic test selection.

## 2. Materials and Methods

### 2.1. Study Design and Ethics

A cross-sectional online survey was conducted. The study was conducted in accordance with the Declaration of Helsinki and approved by the Health and Life Sciences Research Ethics Committee, Aston University, Birmingham, UK (REC IDs: HLS21059 and HLS21060, Version 1, 1 October 2022). Participation was voluntary and fully anonymous; informed consent was obtained via a mandatory consent checkbox preceding the survey instrument.

### 2.2. Participants and Recruitment

Eligible participants were practicing ECPs involved in diagnosing and managing DED, with no restrictions on their country of practice. The survey was hosted on Bristol Online Surveys (Jisc, Bristol, UK) and was active between May 2023 and September 2024. The survey link was distributed electronically through multiple channels: in South Africa, primarily via professional networks, optometry-related social media groups (Facebook and LinkedIn), and through distribution assistance from the South African Optometric Association (SAOA); in the United Kingdom and internationally, via social media platforms. A snowball sampling approach was employed to extend reach within the eye care practitioner community. No personal identifying information was collected.

### 2.3. Survey Instrument

The 32-item English-language questionnaire was developed with reference to TFOS DEWS II guideline domains [3] and adapted from items used in comparable international and regional DED/MGD practice surveys [10,11], supporting content validity by design. The draft questionnaire was piloted by a panel of five practitioners–two expert reviewers and three non-expert reviewers (practicing optometrists)–who completed the questionnaire and assessed content validity comprehension, clarity, completion time and platform functionality prior to launch; this identified one extraneous word in one item, which was removed, and one ambiguously worded item, which was reworded for clarity. Internal consistency of the four-item practitioner confidence subscale (diagnosis, type, severity and management) was excellent (Cronbach’s α = 0.895, *n* = 375 complete cases; item-rest correlations 0.708–0.819). The questionnaire assessed: (i) demographics; (ii) confidence in DED diagnosing, type, severity and management (five-point Likert scale: 1 = not at all confident, 5 = extremely confident); (iii) perceived patient prevalence of DED, MGD, blepharitis and rosacea; (iv) importance of diagnostic tests (Likert 1–5); (v) test-selection criteria via forced-choice and non-restrictive ranking tasks; and (vi) management strategy uptake.

### 2.4. Sample Size

A nominal target of 384 participants was calculated using the conventional single proportion formula for a 95% confidence level and for 5% margin of error (*p* = 0.5). Because recruitment used convenience and snowball sampling rather than probability sampling, this calculation was used as a pragmatic recruitment target rather than as evidence of population-representative precision. Applying finite population correction for SA (about 4000 registered optometrists) would have reduced this target to 351; the more conservative global figure of 384 was retained rather than adopting the lower threshold. For country-level descriptive comparisons, a 10% margin of error was considered acceptable. Using finite population correction, the minimum sample size required based on approximately 4000 registered optometrists was 94 participants; the SA sample exceeded this threshold (*n* = 137). The UK sample (*n* = 112) was considered adequate for exploratory subgroup comparison using the same precision criterion, although population sizes differ between countries.

### 2.5. Statistical Analysis

Data were analysed using IBM SPSS v29 with supplementary verification in Python v3.11. Regional confidence comparisons used two-sided Mann–Whitney U tests; perceived-prevalence and treatment comparisons used chi-square tests of independence. Benjamini–Hochberg FDR correction was applied across all pairwise comparisons (*q*). Effect sizes are reported as Cliff’s delta (δ). Kendall’s tau (τ) assessed rank concordance. Test-selection criteria used both forced-choice and non-restrictive ranking methods; a merged mean rank was computed by averaging the two method means. Statistical significance was set at α = 0.05. No respondents were excluded for incompleteness; analyses used per-item available-case denominators, and item-level missingness across the 32 core questionnaire items ranged from 0% to 3.6% (median 0.5%).

## 3. Results

### 3.1. Participant Demographics

Of 384 respondents, 383 provided sufficient country information for regional classification (representing 31 countries across all six World Health Organisation (WHO) regions and were included in the regional analysis: SA (*n* = 137; 35.8%), the UK (*n* = 112; 29.2%), RoW (*n* = 134; 35.0%). Within the RoW, North America (10.2%), South-East Asia (8.1%) and South America (5.7%) were the largest contributors; the Western Pacific (*n* = 4) and the Eastern Mediterranean (*n* = 15) were sparsely represented. Most participants were optometrists (90.9%), with ophthalmologists (5.2%), ophthalmic nurses and other practitioners (2.9%) and dispensing opticians (1.0%) comprising the remainder; ophthalmologist representation was highest in SA (11.7%). The largest experience groups were 21–30 years (25.0%) and 11–20 years (22.9%). Practitioners with more than 20 years of experience accounted for 41.2% of the sample globally, with a proportionally higher mid- to- late-career representation in SA and the UK than in the RoW (Table 1; see Supplementary Table S1 for the full country-level breakdown).

### 3.2. Practitioner Confidence in DED Diagnosis and Management

**Figure 1:**
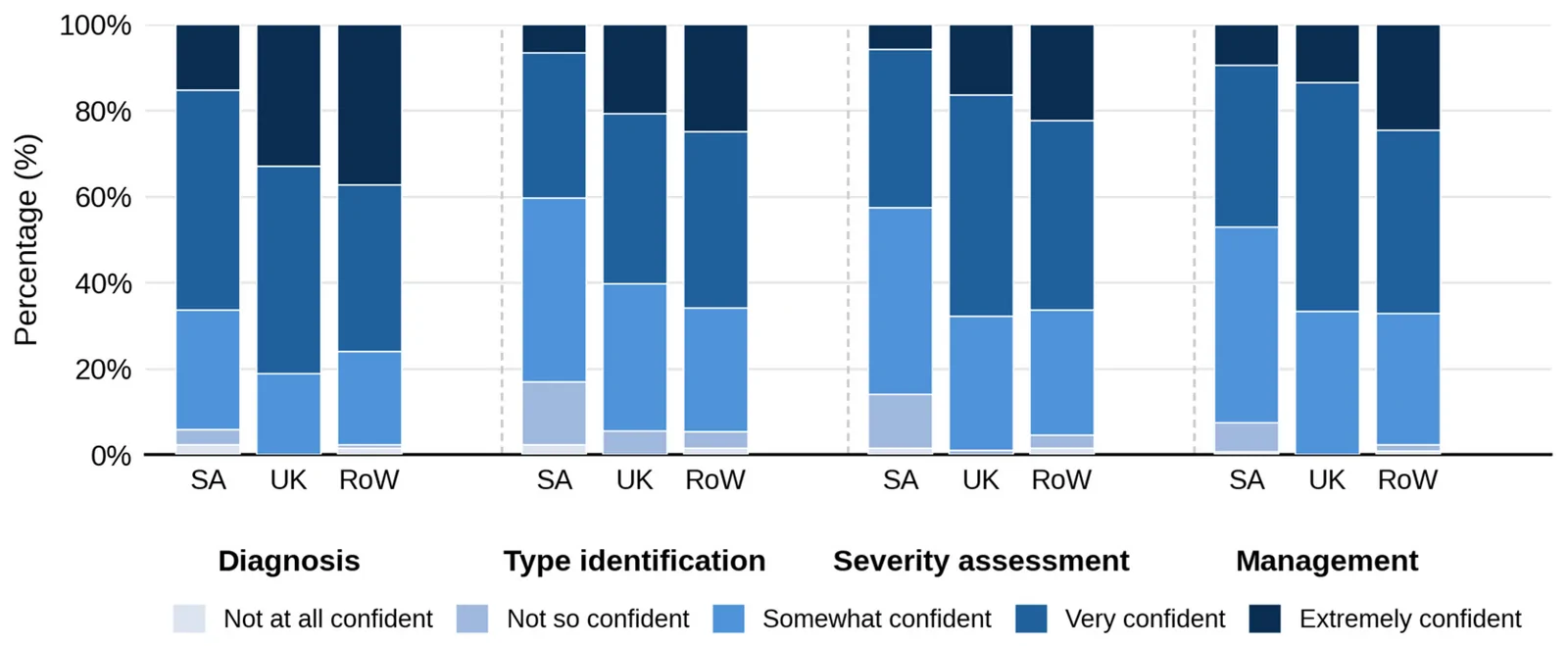
Practitioner confidence across four DED domains by region. Responses are displayed as percentage distributions across five confidence levels. SA = South Africa; UK = United Kingdom; RoW = rest of world.

**Figure 2:**
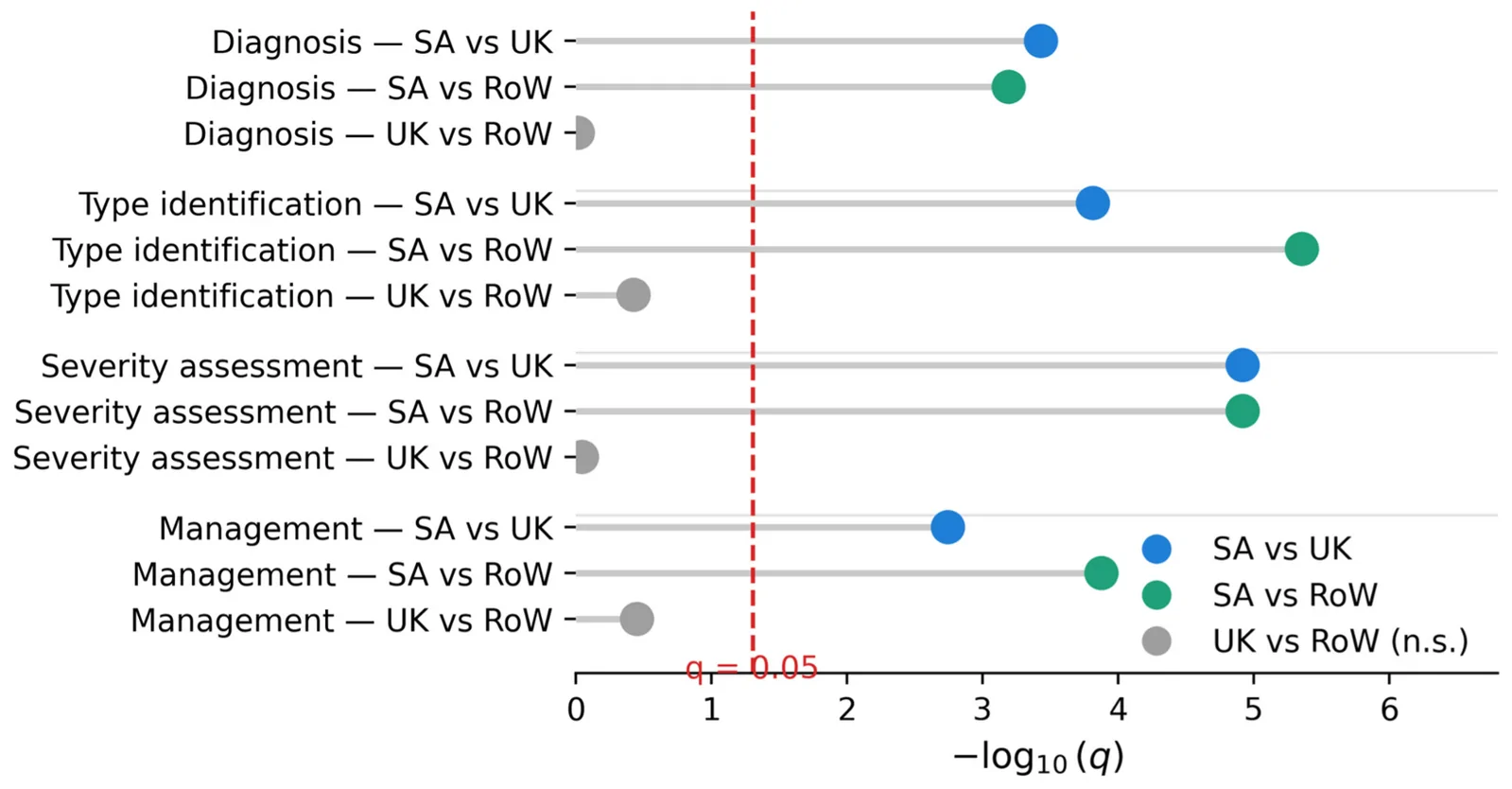
Statistical evidence for between-region confidence differences. Bars show −log_10_(*q*) for each pairwise comparison per domain. Dashed red line marks the significance threshold (−log_10_(0.05) = 1.30). All SA vs. UK and SA vs. RoW comparisons exceed the threshold; UK vs. RoW comparisons do not. SA = South Africa; UK = United Kingdom; RoW = rest of world.

Practitioners globally reported generally high confidence, but SA consistently scored lower than both the UK and RoW in all four domains. After BH-FDR correction, SA differed significantly from the UK in all domains (diagnosis *q* < 0.001, type *q* < 0.001, severity *q* < 0.001, management *q* = 0.003; Cliff’s δ small to medium), and from the RoW in all domains (all *q* < 0.05). UK versus RoW comparisons were non-significant (all *q* ≥ 0.36). Experience positively predicted confidence; practitioners with fewer than five years of experience reported the lowest scores across all regions (Figure 1). Figure 2 shows the statistical evidence for all pairwise comparisons, and Supplementary Table S2 reports 95% confidence intervals for each effect size.

### 3.3. Perceived Prevalence of DED and Associated Conditions

Globally, 70.3% of practitioners reported that ≥41% of their patients had DED, with no significant regional differences. For MGD among DED patients, 57.2% reported this proportion at ≥41%, with a significant difference χ^2^(2) = 6.95, *p* = 0.031); UK practitioners reported significantly higher proportions of anterior blepharitis (SA vs. UK: χ^2^(4) = 28.58, *p* < 0.001, *q* < 0.001; UK vs. RoW: χ^2^(4) = 37.24, *p* < 0.001, *q* < 0.001) and posterior blepharitis (SA vs. UK: χ^2^(4) = 46.57, *p* < 0.001, *q* < 0.001; UK vs. RoW: χ^2^(4) = 36.62, *p* < 0.001, *q* < 0.001) than both SA and RoW. Rosacea recognition differed significantly across all three regional pairs (SA vs. UK: *q* = 0.002; SA vs. RoW: *q* = 0.013; UK vs. RoW: *q* < 0.001), with UK practitioners reporting the highest proportions.

### 3.4. Symptom Profiles

**Figure 3:**
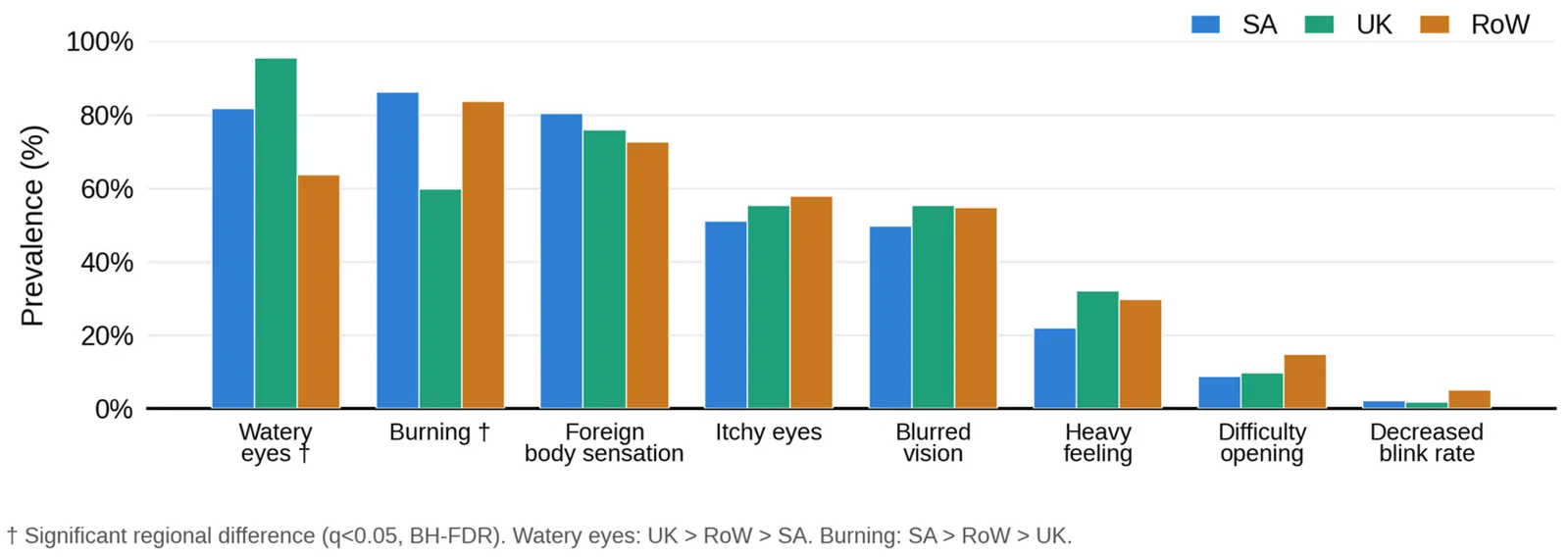
Prevalence of DED symptoms reported by eye care practitioners, by region. ^†^ Significant regional difference after BH-FDR correction. Watery eyes: UK > RoW > SA. Burning: SA > RoW > UK. SA = South Africa; UK = United Kingdom; RoW = rest of world.

The three most commonly reported DED symptoms globally were watery eyes (82.9%), foreign-body sensation (77.1%) and burning (75.3%); the least reported were a decreased blink rate (2.5%) and difficulty keeping eyes open (10.4%). Only watery eyes and burning differed significantly after BH-FDR correction. Watery eyes were most prevalent in the UK (93%) and least in SA (72%); burning was highest in SA (86.1%) and lowest in the UK (59.8%). Symptom ranking was highly concordant across regions (Kendall’s τ = 0.80–0.89; all *p* < 0.001). Figure 3 shows the full symptom profile.

### 3.5. Diagnostic Test Importance for DED (Including MGD-Related Measures)

**Figure 4:**
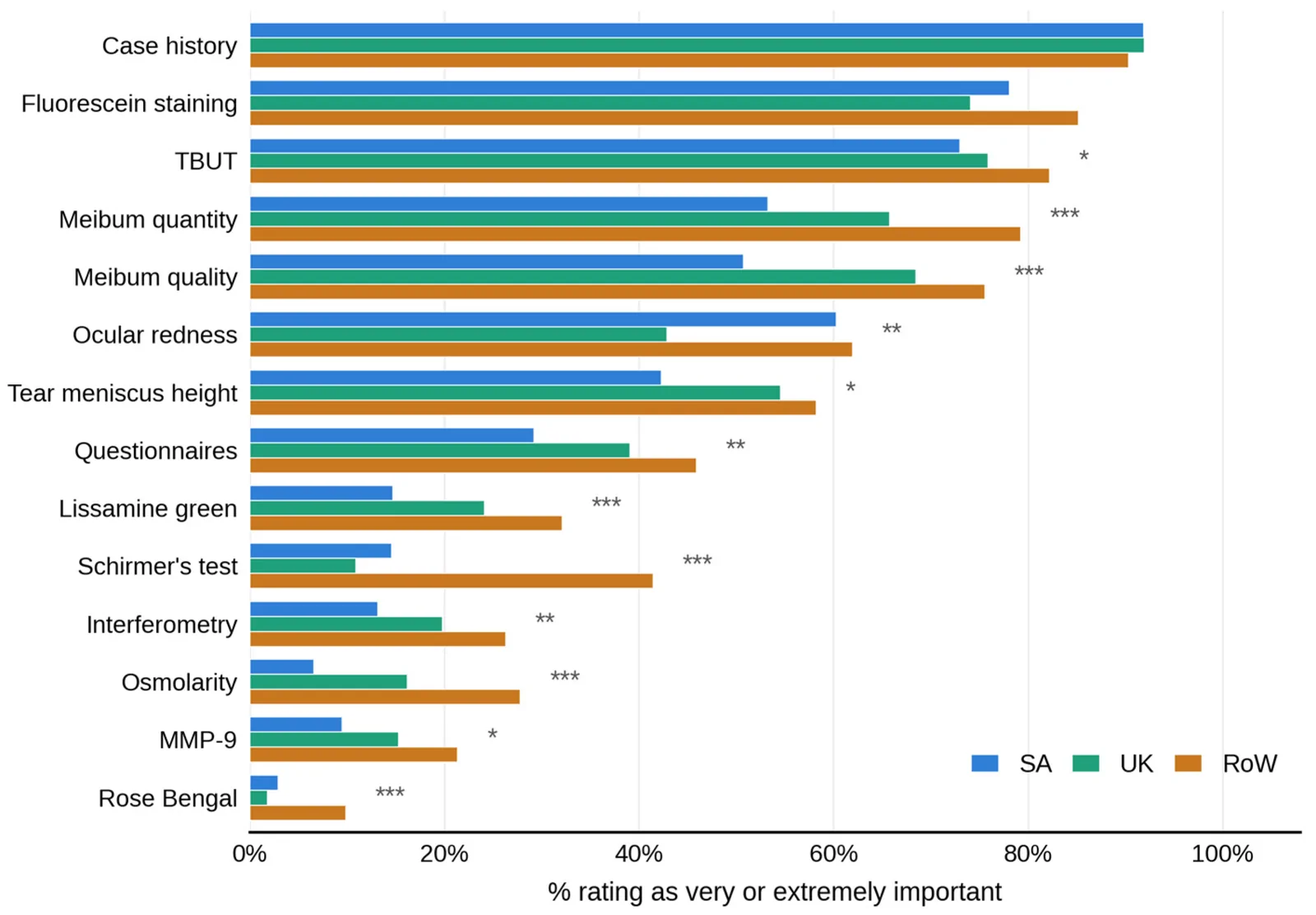
Diagnostic test importance ratings by region. Percentage of respondents rating each test as ‘very’ or ‘extremely’ important (Likert 4–5). Tests are ordered by the global average. Significance indicators reflect BH-FDR corrected Mann–Whitney U tests (* *q* < 0.05, ** *q* < 0.01, *** *q* < 0.001). SA = South Africa; UK = United Kingdom; RoW = rest of world.

Case history was rated as important by 88.7% of practitioners globally, followed by fluorescein staining (79.4%), TBUT (77.3%), meibum quantity (65.8%) and meibum quality (64.5%). Advanced diagnostics—osmolarity, MMP-9 and interferometry—received low global endorsement. Standardised questionnaires were used by 39.4% (OSDI) and 14.4% (SPEED) of practitioners. Figure 4 shows the full comparison across regions. UK practitioners rated osmolarity, meibum quality and tear meniscus height significantly higher than their SA counterparts; SA rated Rose Bengal more highly. RoW practitioners placed greater importance than SA on a broad range of tests.

### 3.6. General Diagnostic Test Selection Criteria (Applicable Across DED and MGD Assessments)

**Figure 5:**
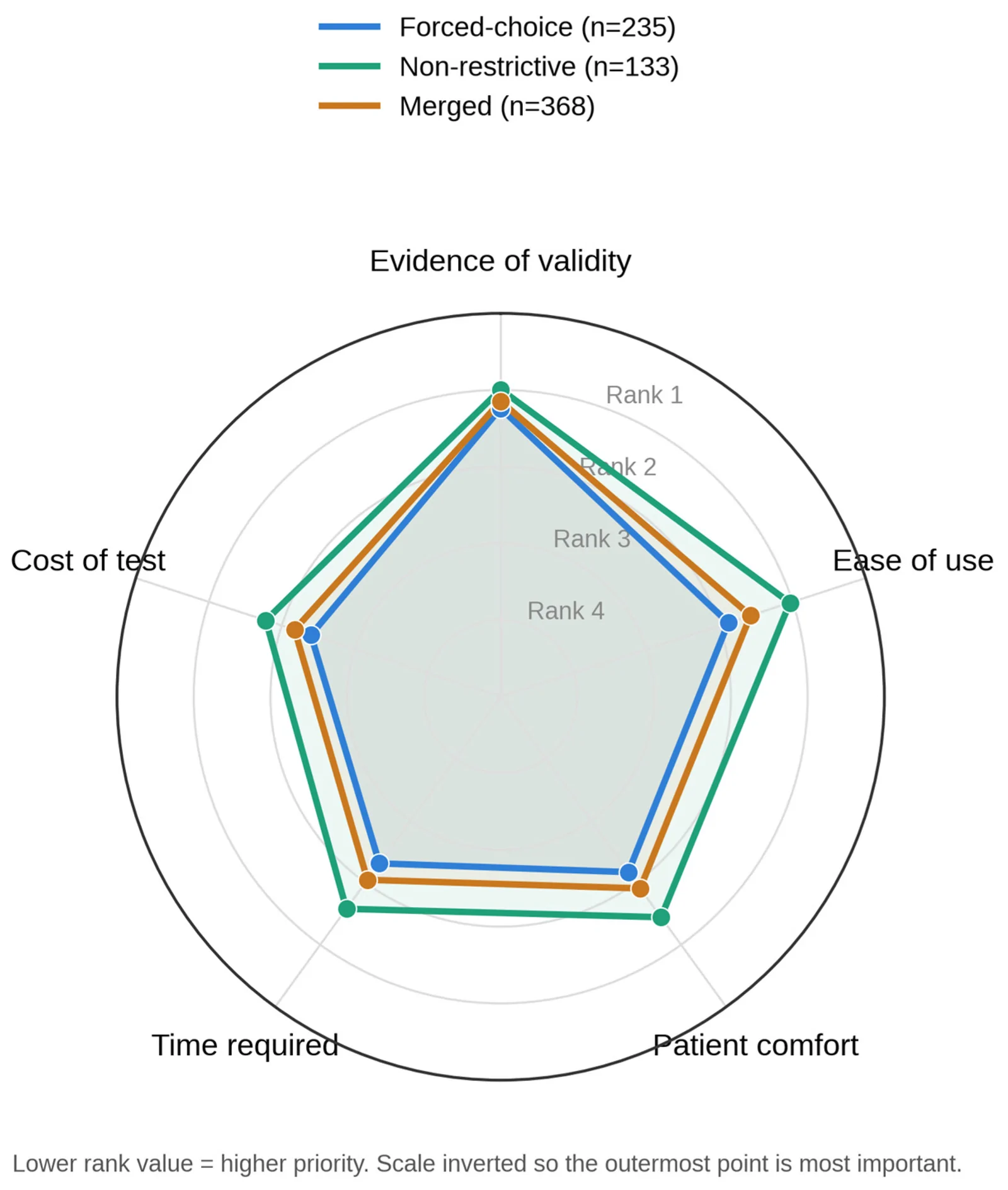
Criteria influencing diagnostic test selection across three ranking methods. Mean rank scores are shown (1 = most important, 5 = least). The radar is inverted so that the innermost point represents the highest priority. Evidence of test validity is consistently ranked first; cost is consistently last.

Evidence of test validity was the top-ranked criterion across all methods (merged mean rank 2.05), followed by ease of use (2.54), patient comfort (2.77), time required (3.07) and cost (3.52). The hierarchy was robust across both ranking methods, as illustrated in Figure 5.

**Figure 6:**
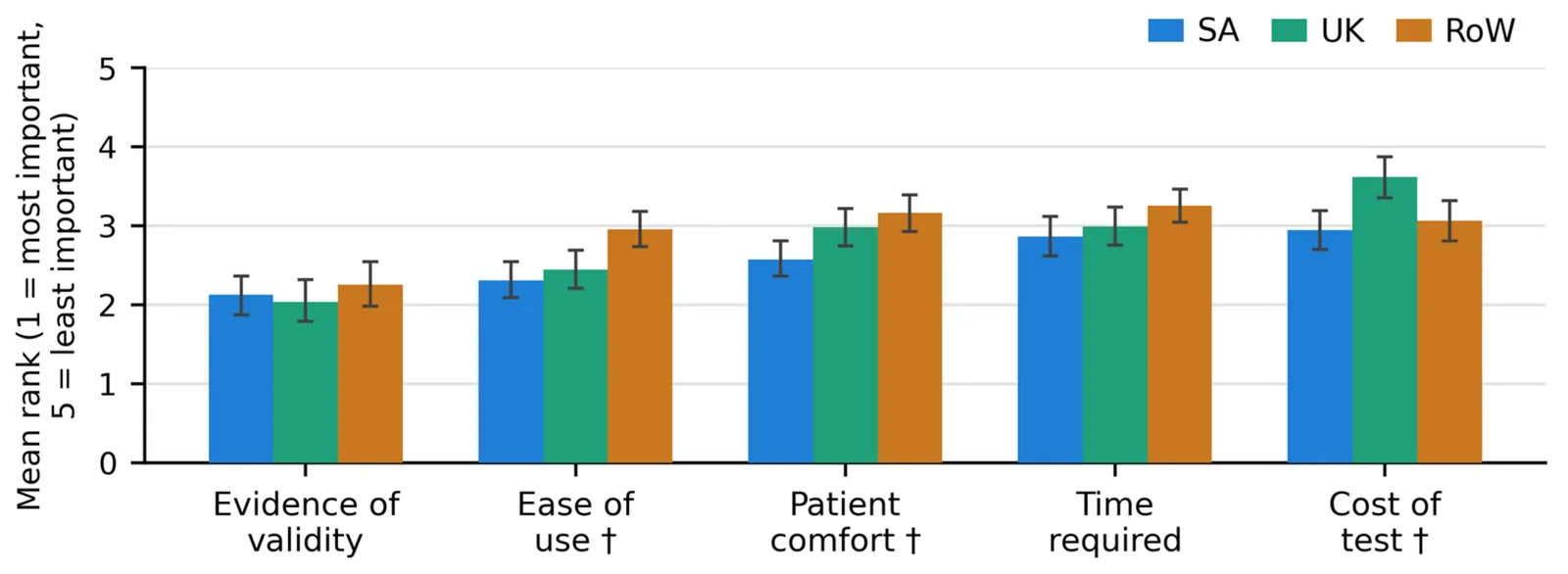
Observed regional differences in diagnostic test-selection criteria priorities. Mean rank shown for SA, UK and RoW (1 = most important, 5 = least important); error bars denote 95% bootstrap confidence intervals. ^†^ Significant regional difference after BH-FDR correction (*q* < 0.05); see Table 2 for full statistics. SA = South Africa; UK = United Kingdom; RoW = rest of world.

Regional comparison of the merged mean ranks (Kruskal–Wallis test with Benjamini–Hochberg FDR-corrected pairwise Mann–Whitney U tests; *n* = 369) showed no significant difference in the priority given to evidence of test validity (*q* = 0.571), indicating universal agreement that this criterion should rank first. In contrast, ease of use, patient comfort and cost differed significantly by region (all omnibus *q* ≤ 0.018): SA and UK practitioners prioritised ease of use and patient comfort more highly than RoW (all *q* < 0.03), while UK practitioners deprioritised cost relative to both SA and RoW (*q* < 0.03). Time required showed a similar but non-significant trend (*q* = 0.068). Figure 6 and Table 2 summarise these regional comparisons; full pairwise statistics are provided in Supplementary Table S3.

### 3.7. Treatment Practices for DED and MGD

**Figure 7:**
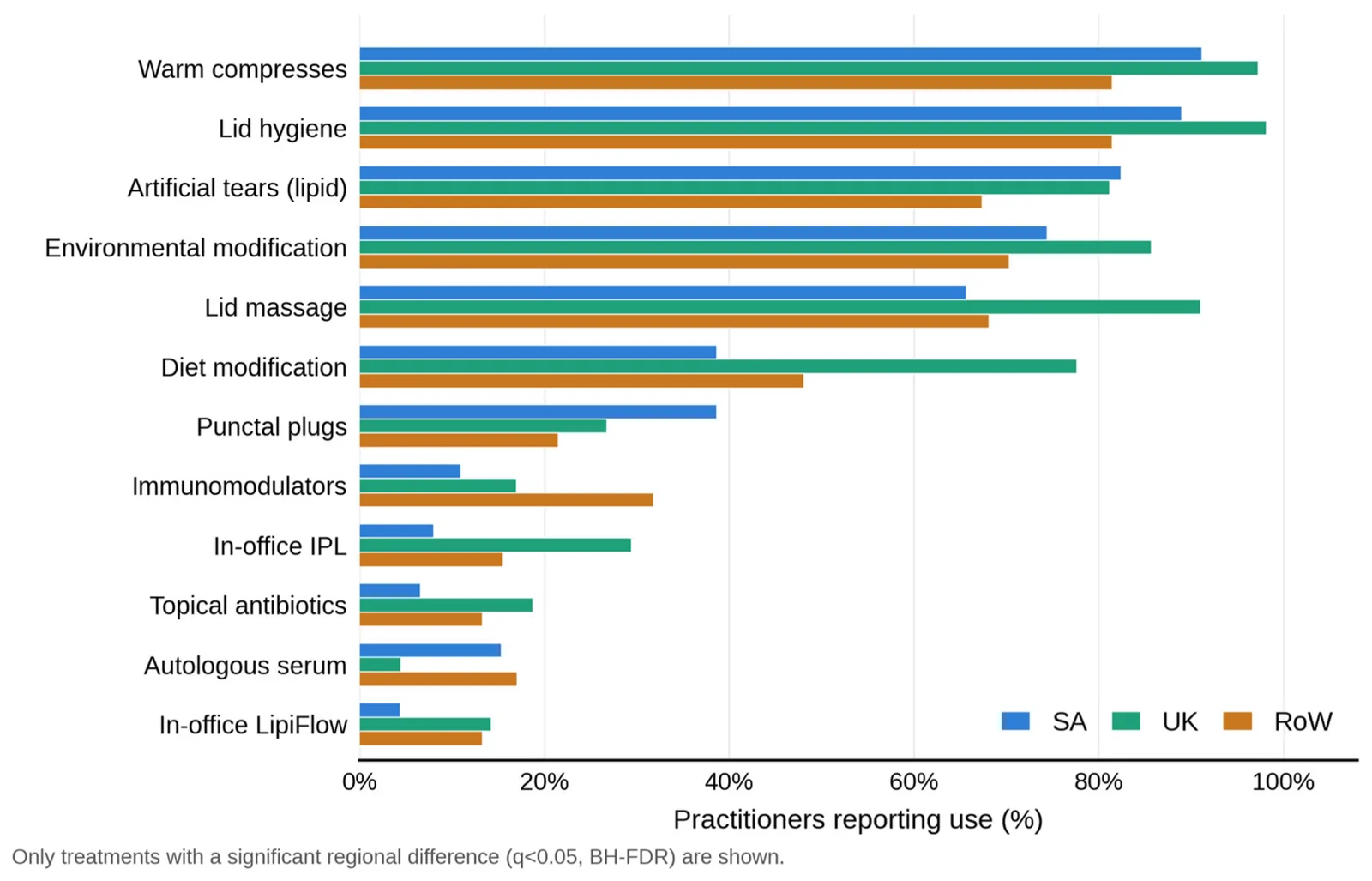
Regional treatment utilisation by SA, UK and RoW. Only treatments with a significant regional difference after BH-FDR correction (*q* < 0.05) are shown. Table 3 provides full statistical details. SA = South Africa; UK = United Kingdom; RoW = rest of world.

Lid hygiene (89.3%) and warm compresses (89.6%) were the most commonly recommended interventions globally, followed by artificial tears (77.0%) and omega-3 supplementation (62.0%). UK practitioners reported significantly higher use of lid hygiene, warm compresses, lid massage, dietary modification, environmental modification and in-office IPL compared with both SA and the RoW (all *q* < 0.05), with in-office LipiFlow^®^ (Johnson & Johnson Vision, Santa Ana, CA, USA) also higher in the UK than in SA (*q* = 0.046). RoW practitioners reported significantly higher use of immunomodulatory drugs than both SA (*q* < 0.001) and the UK (*q* = 0.046). SA practitioners reported higher use of punctal plugs than RoW (*q* = 0.023) and higher use of lipid-containing artificial tears than RoW (*q* = 0.034). Both SA and RoW reported higher use of autologous serum than the UK (SA vs. UK: *p* = 0.010, *q* = 0.043; RoW vs. UK: *q* = 0.025). Oral antibiotics did not differ significantly between regions after BH-FDR correction. Despite these regional differences in uptake rates, Kendall’s τ concordance (τ = 0.73–0.85; all *p* < 0.0001) confirmed strong agreement in the relative ranking of management strategies across regions. Figure 7 displays regional utilisation rates for treatments with significant differences. Table 3 provides complete chi-square statistics.

## 4. Discussion

This study provides a comparative analysis of DED diagnosis and management, with additional data on perceived MGD prevalence, diagnostic emphasis and treatment uptake across SA, the UK and a global RoW cohort, drawing on data from 384 ECPs in 31 countries. The findings confirm a recognisable international framework for DED care, with additional insights into MGD-related practice, while revealing substantial regional disparities in practitioner confidence, diagnostic emphasis and therapeutic access.

South African practitioners reported significantly lower confidence across all four domains, with small-to-medium effect sizes that were robust to multiple testing correction. Confidence is a subjective self-assessment and was not benchmarked against objective measures of diagnostic accuracy or patient outcomes in this study; lower confidence should not be interpreted as evidence of reduced clinical competence, as clinical competence was not objectively assessed in this study. The positive relationship between experience and confidence aligns with established evidence that clinical self-efficacy develops progressively with supervised practice and an expanding scope [13]. This underscores the importance of structured mentorship and accessible CPD early in the career.

Structural factors are likely to contribute to the confidence gap. The UK has more than 1700 therapeutic optometrists able to prescribe independently [14], whereas only approximately 27 practitioners in SA currently hold therapeutic certification [15]. Differences in prescribing scope may be one of several contextual factors associated with differences in reported confidence; however, the present study cannot determine whether prescribing scope contributed to the observed confidence differences.

The primacy of case history, fluorescein TBUT and ocular surface staining was consistent globally, reflecting established accessibility and clinical utility. The declining status of Schirmer’s test reflects longstanding concerns about reproducibility and patient discomfort [16,17]. The relatively low reported use of osmolarity and MMP-9 testing, despite their endorsement in TFOS DEWS II, and continued endorsement in the more recent TFOS DEWS III, may reflect a combination of cost, accessibility, equipment availability, practitioner training and local clinical practice patterns. However, these factors were not directly assessed in the present survey and their relative contributions cannot be determined. A dedicated barrier-attribution item would strengthen future survey iterations. Notably, this pattern sits in tension with practitioners’ own stated selection criteria: evidence of test validity was rated the most important criterion for test selection overall, while cost was rated as the least important (Figure 5), yet uptake of these validated, evidence-based tests remains low. This divergence between stated priorities and observed practice may itself reflect the self-reported nature of the data or may indicate that cost and access act as practical constraints that override stated preferences when practitioners are not explicitly reasoning through the trade-off.

**Figure 8:**
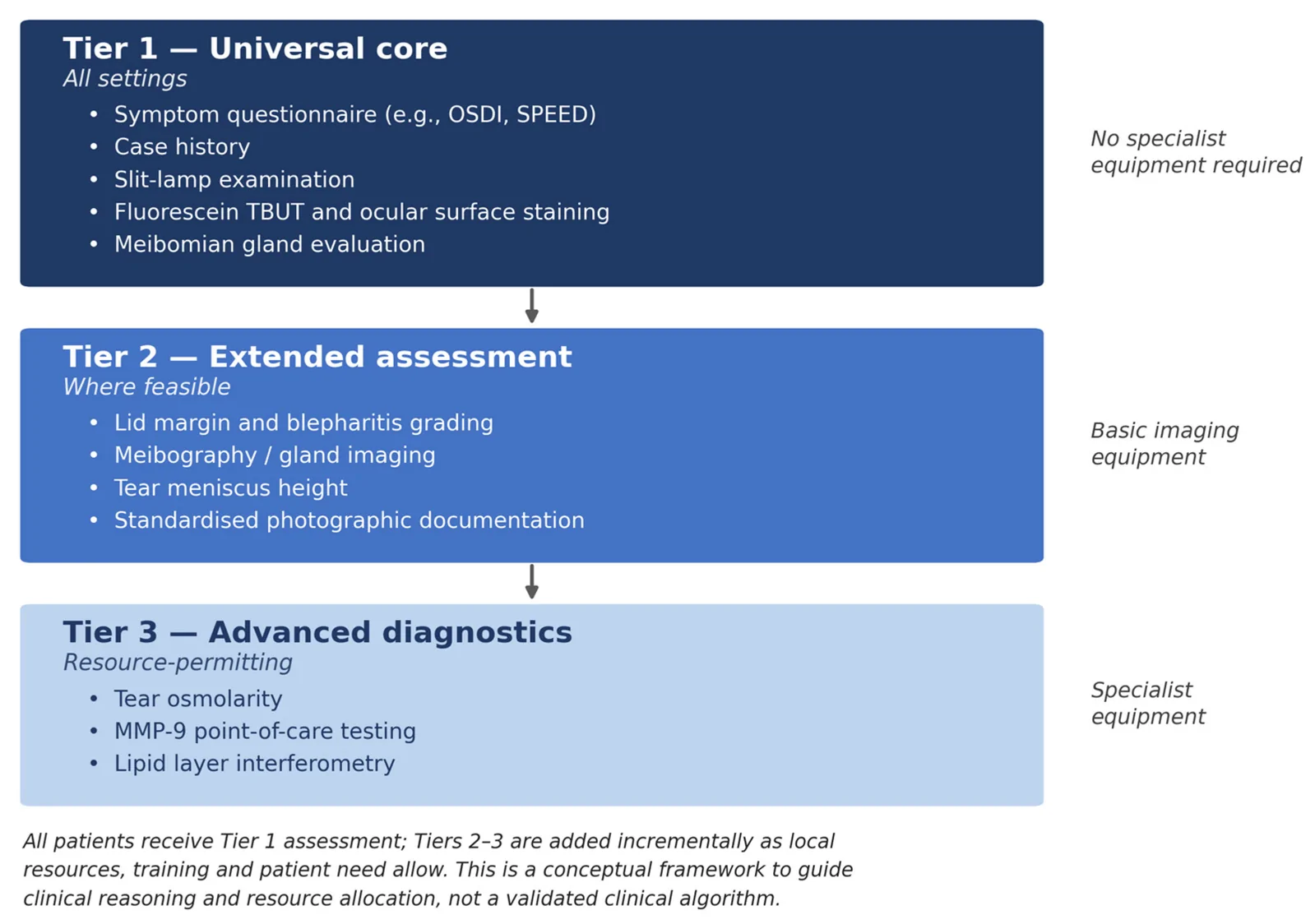
Proposed tiered diagnostic framework for dry eye disease. Tier 1 (universal core) is proposed as a minimum assessment framework for consideration across settings; Tiers 2 and 3 are added incrementally as local resources, training and patient need allow. The framework is a conceptual model derived from the study’s findings and is not a validated clinical algorithm.

The UK practitioners’ higher ratings for osmolarity, meibum quality and tear meniscus height may reflect differences in clinical training, professional practice patterns or access to diagnostic technologies. SA’s diagnostic repertoire is narrower than those of both the UK and the RoW—reflected also in a lower recognition of anterior and posterior blepharitis. The underendorsement of standardised questionnaires (e.g., OSDI) in SA is a missed opportunity: these tools are low-cost, patient-centred and well-validated. A tiered diagnostic framework is proposed: a universal core (case history, symptom questionnaire, slit-lamp examination with TBUT and meibomian gland evaluation) supplemented by advanced tools (osmolarity, MMP-9, interferometry) where resources permit. Figure 8 illustrates this proposed framework schematically. It is presented as a conceptual framework intended to guide clinical reasoning and resource allocation, not as a validated clinical algorithm; its diagnostic accuracy, feasibility and impact on patient outcomes have not been tested and require prospective evaluation before adoption into formal practice guidelines.

The broader pattern of significantly higher UK uptake across lid hygiene, warm compresses, lid massage, dietary modification, environmental modification and in-office therapies (IPL and LipiFlow^®^) reflects both greater therapeutic prescribing scope and better technology access in the UK compared with SA and RoW. The higher utilisation of autologous serum in both SA and RoW compared with the UK likely reflects lower uptake in the UK context, where prescribing constraints and formulary restrictions may limit access, rather than unusually high uptake in other regions. These findings warrant further prospective investigation. Despite these regional differences in uptake rates, Kendall’s τ concordance (τ = 0.73–0.85) confirmed that the relative ranking of management strategies is broadly shared internationally. Beyond prescribing scope and practitioner preference, broader healthcare-system characteristics are also likely to contribute to these regional differences, including reimbursement structures, insurance or formulary access, and the capital cost of in-office equipment such as IPL and LipiFlow^®^ devices. Disentangling the relative contributions of practitioner preference, structural constraints and economic factors was beyond the scope of the present survey and represents an important direction for future research. These findings can also be contextualised against prior international surveys. The 2021 TFOS International Survey recruited a larger, more multidisciplinary sample (1139 practitioners from 51 countries; 58% optometrists and 37% ophthalmologists) [10] than the present study, in which 90.9% of participants were optometrists. This difference limits the direct comparability of prescribing-related findings between the two surveys. Nevertheless, Downie et al.’s Australia–UK comparison (654 Australian and 1006 UK optometrists) [11] and Casemore et al.’s UK optometrist practice-pattern survey [9] similarly reported higher UK therapeutic uptake associated with expanded independent-prescribing rights, consistent with the pattern observed here (Supplementary Table S4).

Strengths include the broad international scope of the study (384 practitioners from 31 countries across six WHO regions), the direct comparison of SA and the UK within the same dataset, rigorous BH-FDR correction and the use of dual ranking methods. Several limitations should be considered. Recruitment relied on professional networks and social media rather than random sampling from professional registers, which is susceptible to selection bias. Practitioners who are more professionally active or digitally engaged, particularly in SA, may be over-represented and may also be more likely to have higher CPD engagement; this could inflate reported confidence and guideline-adherent practice relative to the wider ECP population. Relatedly, because participation was voluntary, non-response bias cannot be excluded: practitioners with lower interest or confidence in DED management may have been less likely to complete the survey, which could further inflate reported confidence scores. Findings should therefore be interpreted as representative of professionally engaged, connected practitioners rather than all practising ECPs, and future studies using random sampling from professional registers would strengthen generalizability. A true response rate could not be calculated: the survey was distributed via open social media and professional-network posts with a snowball component and no closed sampling frame or fixed invitee list, so the number of practitioners who viewed or received the survey link is unknown. A manual check for duplicate submissions, matching the completion date, city, profession and years of experience, identified only two matched pairs (four respondents) among the 384 analysable responses (<1.5%); however, this check was necessarily conservative, as completion dates were missing for 35.7% of respondents and the anonymous survey design did not incorporate a technical duplicate-submission safeguard. All outcomes—perceived disease prevalence, diagnostic test importance and treatment uptake—are self-reported estimates rather than data drawn from audited clinical records, so actual clinical practice may differ from what is reported here; social desirability bias may particularly inflate self-reported adherence to guideline-recommended core diagnostic testing. Further limitations include: (i) the disproportionate representation of SA and the UK, with sparse Western Pacific and Eastern Mediterranean data—the RoW category in particular aggregates markedly heterogeneous regions, including North America, South-East Asia, South America, and sparsely represented Western Pacific and Eastern Mediterranean groups, so meaningful within-RoW regional differences may be obscured; (ii) the predominance of optometrists, who comprised 90.9% of the sample, limiting generalisability to multidisciplinary practice and to ophthalmologists in particular; (iii) the predominance of mid- to late-career participants; and (iv) the use of an English-language survey (Supplementary Table S5).

## 5. Conclusions

This international survey documents a shared global foundation for DED care, together with region-specific patterns in MGD-related diagnostic and treatment practices, and meaningful regional disparities in confidence, diagnostic priorities and treatment access. Core diagnostic approaches are internationally consistent, while advanced diagnostics received low reported importance globally. These findings reflect practitioners’ self-reported perceptions and confidence and should not be assumed to directly correspond to objectively measured clinical practice or patient-level outcomes.

A tiered diagnostic framework is proposed, applying a universal core supplemented by advanced tools where resources permit. Future research should expand representation from underserved regions, link practitioner confidence to measurable patient outcomes, and test the feasibility of tiered care protocols across diverse healthcare settings.

## Figures and Tables

**Table 1:** Practitioner demographics by region.

Characteristic	Global (*n* = 384)	SA (*n* = 137)	UK (*n* = 112)	RoW (*n* = 134)
**Profession**				
Optometrist	349 (90.9%)	116 (84.7%)	104 (92.9%)	128 (95.5%)
Ophthalmologist	20 (5.2%)	16 (11.7%)	1 (0.9%)	3 (2.2%)
Dispensing optician	4 (1.0%)	0 (0.0%)	3 (2.7%)	1 (0.7%)
Other/Ophthalmic nurse	11 (2.9%)	5 (3.6%)	4 (3.6%)	2 (1.5%)
**Years in practice**				
Less than 1 year	13 (3.4%)	8 (5.8%)	1 (0.9%)	4 (3.0%)
1–5	64 (16.7%)	23 (16.8%)	14 (12.5%)	27 (20.1%)
6–10	57 (14.8%)	12 (8.8%)	18 (16.1%)	27 (20.1%)
11–20	88 (22.9%)	28 (20.4%)	23 (20.5%)	37 (27.6%)
21–30	96 (25.0%)	39 (28.5%)	33 (29.5%)	23 (17.2%)
31–40	49 (12.8%)	18 (13.1%)	18 (16.1%)	13 (9.7%)
41–50	15 (3.9%)	7 (5.1%)	5 (4.5%)	3 (2.2%)
51–60 ^†^	2 (0.5%)	2 (1.5%)	0 (0.0%)	0 (0.0%)
**WHO Region**				
Africa	146 (38.1%)	137 (100.0%)	0 (0.0%)	9 (6.7%)
Europe	126 (32.9%)	0 (0.0%)	112 (100.0%)	14 (10.4%)
North America	39 (10.2%)	0 (0.0%)	0 (0.0%)	39 (29.1%)
South-East Asia	31 (8.1%)	0 (0.0%)	0 (0.0%)	31 (23.1%)
South America	22 (5.7%)	0 (0.0%)	0 (0.0%)	22 (16.4%)
Eastern Mediterranean	15 (3.9%)	0 (0.0%)	0 (0.0%)	15 (11.2%)
Western Pacific	4 (1.0%)	0 (0.0%)	0 (0.0%)	4 (3.0%)

^†^ One participant did not complete the country field and was excluded from regional analyses (*n* = 383); their profession and years-in-practice responses were complete and are retained above (*n* = 384).

**Table 2:** Observed regional differences in diagnostic test-selection criteria priorities (Kruskal–Wallis omnibus test, Benjamini–Hochberg FDR corrected).

Criterion	SA	UK	RoW	Omnibus *q* (BH-FDR)	Significant Pairwise Differences (*q* < 0.05)
Evidence of validity	2.13	2.04	2.25	0.571	None
Ease of use	2.31	2.44	2.95	<0.001	SA vs. RoW (*q* = 0.001); UK vs. RoW (*q* = 0.011)
Patient comfort	2.57	2.98	3.16	0.002	SA vs. UK (*q* = 0.029); SA vs. RoW (*q* = 0.002)
Time required	2.86	2.99	3.25	0.068	None
Cost	2.94	3.61	3.06	0.002	SA vs. UK (*q* = 0.002); UK vs. RoW (*q* = 0.011)

**Table 3:** Significant regional differences in treatment utilisation after Benjamini–Hochberg FDR correction.

Treatment	Comparison	Group 1 (%)	Group 2 (%)	χ^2^	df	*p*	*q* (BH-FDR)	Higher in
Diet modification	SA vs. UK	38.7%	77.7%	38.07	1	<0.001	<0.001	UK
Diet modification	UK vs. RoW	77.7%	48.5%	21.99	1	<0.001	<0.001	UK
Lid massage	UK vs. RoW	91.1%	67.9%	19.36	1	<0.001	<0.001	UK
In-office IPL	SA vs. UK	8.0%	29.5%	19.46	1	<0.001	<0.001	UK
Lid massage	SA vs. UK	65.7%	91.1%	22.48	1	<0.001	<0.001	UK
Immunomodulatory drugs	SA vs. RoW	10.9%	32.1%	18.0	1	<0.001	<0.001	RoW
Lid hygiene	UK vs. RoW	98.2%	82.1%	16.78	1	<0.001	<0.001	UK
Warm compresses	UK vs. RoW	97.3%	81.3%	15.44	1	<0.001	<0.001	UK
Autologous serum	UK vs. RoW	4.5%	17.2%	9.76	1	0.002	0.013	RoW
Punctal plugs	SA vs. RoW	38.7%	21.6%	9.33	1	0.002	0.015	SA
Topical antibiotics	SA vs. UK	6.6%	18.8%	8.63	1	0.003	0.020	UK
Environmental modification	UK vs. RoW	85.7%	70.1%	8.41	1	0.004	0.021	UK
Lid hygiene	SA vs. UK	89.1%	98.2%	8.13	1	0.004	0.022	UK
Autologous serum	SA vs. UK	15.3%	4.5%	7.78	1	0.005	0.024	SA
Lipid-containing artificial tears	SA vs. RoW	82.5%	67.9%	7.73	1	0.005	0.024	SA
Immunomodulatory drugs	UK vs. RoW	17.0%	32.1%	7.4	1	0.006	0.025	RoW
In-office combined warmth/compression (e.g., LipiFlow^®^)	SA vs. UK	4.4%	14.3%	7.51	1	0.006	0.025	UK
In-office combined warmth/compression (e.g., LipiFlow^®^)	SA vs. RoW	4.4%	13.4%	6.88	1	0.009	0.032	RoW
In-office IPL	UK vs. RoW	29.5%	15.7%	6.77	1	0.009	0.032	UK

## Data Availability

The data presented in this study are available on request from the corresponding author. Data are not publicly available due to participant privacy restrictions in accordance with the study ethics approval.

## Supplementary Materials

The following supporting information can be downloaded at: https://www.mdpi.com/article/10.3390/jcm15176757/s1, Table S1: Respondents by country and WHO region; Table S2: 95% confidence intervals for practitioner-confidence effect sizes; Table S3: Full pairwise regional comparisons for diagnostic test-selection criteria (Kruskal–Wallis/Mann–Whitney U, BH-FDR corrected); Table S4: Comparison with prior international and regional dry eye practice-pattern surveys; Table S5: Study limitations; Implications for Practice and Policy.

## Abbreviations

The following abbreviations are used in this manuscript:

BH-FDR	Benjamini–Hochberg false discovery rate
CPD	Continuing professional development
DED	Dry eye disease
ECP	Eye care practitioner
IPL	Intense pulsed light
MGD	Meibomian gland dysfunction
MMP-9	Matrix metalloproteinase-9
OSDI	Ocular Surface Disease Index
RoW	Rest of world
SA	South Africa
SAOA	South African Optometric Association
SPEED	Standard Patient Evaluation of Eye Dryness
TBUT	Tear break-up time
TFOS DEWS	Tear Film and Ocular Surface Society Dry Eye Workshop
UK	United Kingdom
