# Supplementary material for: International Survey of Eye Care Practitioners on Dry Eye Disease: A Comparative Analysis of South Africa, the United Kingdom and Other Global Regions
Source: J Clin Med. 2026 Aug 31;15(17):6757. doi: 10.3390/jcm15176757 (PMC13566151; doi:10.3390/jcm15176757)
Supplement: Supplementary file 1 [file jcm-15-06757-s001.zip › jcm-4490103-supplementary.pdf]

Supplementary Materials

International Survey of Eye Care Practitioners on Dry Eye Disease: A Comparative Analysis of South Africa, the United Kingdom and Other Global Regions

Christine Croker, Shehzad A. Naroo and Raquel Gil-Cazorla

Table S1. Respondents by country and WHO region.

WHO Region	Country	n	%
Africa	South Africa	137	35.8
Africa	Namibia	7	1.8
Africa	Ghana	1	0.3
Africa	Kenya	1	0.3
Eastern Mediterranean	Lebanon	5	1.3
Eastern Mediterranean	Pakistan	3	0.8
Eastern Mediterranean	Palestine	2	0.5
Eastern Mediterranean	United Arab Emirates (UAE)	2	0.5
Eastern Mediterranean	Iran	1	0.3
Eastern Mediterranean	Iraq	1	0.3
Eastern Mediterranean	Saudi Arabia	1	0.3
Europe	United Kingdom (UK)	112	29.2
Europe	Ireland	4	1.0
Europe	Spain	4	1.0
Europe	Italy	2	0.5
Europe	Croatia	1	0.3
Europe	France	1	0.3
Europe	Greece	1	0.3
Europe	Switzerland	1	0.3
North America	United States of America (USA)	20	5.2
North America	Trinidad and Tobago	14	3.7
North America	Guatemala	3	0.8
North America	Canada	2	0.5
South America	Colombia	19	5.0
South America	Argentina	1	0.3
South America	Ecuador	1	0.3
South America	Peru	1	0.3
South-East Asia	India	29	7.6
South-East Asia	Nepal	2	0.5
Western Pacific	Malaysia	2	0.5
Western Pacific	New Zealand	2	0.5

Computed directly from the raw survey dataset. n = 383; one respondent did not complete the country field and is excluded (see main text, Table 1 footnote). Countries are grouped by World Health Organisation (WHO) region and ordered by region, then by descending respondent count. SA = South Africa; UK = United Kingdom.

Table S2. 95% confidence intervals for practitioner-confidence effect sizes.

Domain	Comparison	n_1	n_2	Cliff's δ	CI low	CI high
Diagnosis	SA vs UK	137	112	-0.254	-0.386	-0.126
Diagnosis	SA vs RoW	137	133	-0.235	-0.356	-0.104
Type	SA vs UK	136	111	-0.279	-0.405	-0.147
Type	SA vs RoW	136	131	-0.341	-0.454	-0.22
Severity	SA vs UK	136	109	-0.323	-0.437	-0.202
Severity	SA vs RoW	136	133	-0.312	-0.436	-0.194
Management	SA vs UK	136	111	-0.22	-0.344	-0.085
Management	SA vs RoW	136	133	-0.27	-0.389	-0.141

Cliff's delta (δ) with bootstrap 95% confidence intervals (2,000 resamples), computed directly from the raw survey dataset for the four practitioner-confidence domains reported in Section 3.2 of the main text. Negative values indicate South Africa (SA) scored lower than the comparator region. n_1 and n_2 denote the number of respondents with non-missing data in each compared group. UK = United Kingdom; RoW = rest of world.

Table S3. Full pairwise regional comparisons for diagnostic test-selection criteria priorities.

Criterion	Comparison	$Mean_1$	$Mean_2$	p	q (BH-FDR)
Evidence of validity	SA vs RoW	2.13	2.25	0.650	0.650
Evidence of validity	SA vs UK	2.13	2.04	0.538	0.576
Evidence of validity	UK vs RoW	2.04	2.25	0.285	0.423
Ease of use	SA vs RoW	2.31	2.95	<0.001	<0.001
Ease of use	SA vs UK	2.31	2.44	0.310	0.423
Ease of use	UK vs RoW	2.44	2.95	0.003	0.011
Patient comfort	SA vs RoW	2.57	3.16	<0.001	0.002
Patient comfort	SA vs UK	2.57	2.98	0.011	0.029
Patient comfort	UK vs RoW	2.98	3.16	0.271	0.423
Time required	SA vs RoW	2.86	3.25	0.024	0.051
Time required	SA vs UK	2.86	2.99	0.412	0.515
Time required	UK vs RoW	2.99	3.25	0.095	0.179
Cost	SA vs RoW	2.94	3.06	0.502	0.576
Cost	SA vs UK	2.94	3.61	<0.001	0.002
Cost	UK vs RoW	3.61	3.06	0.004	0.011

Pairwise Mann-Whitney U tests (two-sided) with Benjamini-Hochberg FDR correction, computed directly from the raw survey dataset for the five test-selection criteria reported in Section 3.6 of the main text (merged forced-choice/non-restrictive ranking method; n=369). $Mean_1$ and $Mean_2$ are the mean ranks (1 = most important, 5 = least important) for the first- and second-named region in each comparison. SA = South Africa; UK = United Kingdom; RoW = rest of world. See main-text Table 2 for the omnibus (Kruskal-Wallis) test per criterion and Figure 6 for a graphical summary.

Table S4. Comparison with prior international and regional dry eye practice-pattern surveys.

Study	Sample	Scope	Fluorescein staining	TBUT	Osmolarity uptake	Key comparative note
This study (2026)	n=383 (31 countries)	SA / UK / RoW comparison	79.4%	77.1%	16.8%†	First direct SA–UK comparison; regional gradient in advanced-test uptake
TFOS International Survey 2021 [10]	n=1,139 (51 countries)	Global, multidisciplinary (58% optom., 37% ophth.)	Not reported	Not reported	Not reported	Largest, most multidisciplinary comparator sample
Downie et al. 2016 [11]	AUS n=654; UK n=1,006	Australia–UK optometrists	Not reported	Not reported	Not reported	Two-region design; UK led therapeutic uptake
Casemore et al. 2023 [9]	n=131	UK optometrists only	Among most-used	Among most-used	Not specifically reported	18.7% held Independent Prescriber status
Cartes et al. 2023 [8]	n=77 of 109 invited (70.6% response)	International DED experts (N. America 27%, Europe 40%)	92%	Evaluated (highest importance in Europe)	66.2%	Expert-only sample; osmolarity uptake ~4× higher than general-practitioner samples

† Percentage of respondents rating osmolarity as 'very' or 'extremely' important for diagnosis (Likert 4–5), rather than confirmed test use; regional breakdown: SA 6.6%, UK 16.2%, RoW 28.0%. 'Not reported' indicates the metric was not extractable from the original publication's abstract or results as accessed, not that it is absent from the underlying study.

Strengths include broad international scope (383 practitioners, 31 countries, 6 WHO regions), direct comparison of SA and UK within the same dataset, rigorous BH-FDR correction, and dual ranking methods. Several limitations should be considered, summarised in Table 5. Most centrally, convenience/snowball recruitment via professional networks and social media likely over-represents professionally active, digitally engaged practitioners, and voluntary participation raises the possibility of non-response bias; both could inflate reported confidence relative to the wider ECP population. All outcomes are self-reported rather than drawn from audited clinical records, and sample composition – recruitment channel, professional mix, career stage, regional balance and survey language – further constrains generalisability, as detailed in Table 5.

Table S5. Study limitations.

Limitation	Detail	Implication
Recruitment method	Convenience/snowball sampling via professional networks and social media; no random sampling from professional registers.	May over-represent professionally active, digitally engaged practitioners (particularly in SA); could inflate reported confidence and guideline-adherent practice.
Non-response bias	Voluntary participation; a true response rate could not be calculated (open distribution, no fixed invitee list).	Practitioners with lower DED interest/confidence may be under-represented, further inflating reported confidence scores.
Duplicate submissions	Manual check found 2 matched pairs (4 respondents) of 383 (<1.5%); conservative, as completion dates were missing for 35.7% of respondents; no technical safeguard was in place.	Residual duplication cannot be fully excluded.
Self-reported outcomes	All prevalence, diagnostic-importance and treatment data are practitioner-reported, not drawn from audited clinical records.	Actual clinical practice may differ; social desirability bias may inflate reported adherence to guideline-recommended testing.
Regional sample balance	SA and UK over-represented; Western Pacific and Eastern Mediterranean sparse; RoW aggregates heterogeneous regions.	Within-RoW regional differences may be obscured.
Professional mix	Optometrists comprised 90.9% of respondents.	Limits generalisability to multidisciplinary practice and to ophthalmologists specifically.
Career stage	Most participants were mid-to-late career.	Findings may not generalise to early-career practitioners.
Survey language	Conducted in English only.	May have excluded eligible non-English-speaking practitioners, particularly outside SA/UK.

Implications for Practice and Policy

These findings point to specific, actionable steps for stakeholders positioned to close the disparities identified. Professional bodies (e.g., the South African Optometric Association, the College of Optometrists) should prioritise accessible, low-cost continuing professional development in advanced DED/MGD diagnostics, targeted at settings where confidence and diagnostic-test uptake are lowest. Regulators reviewing scope-of-practice frameworks should consider the confidence and treatment-access gap associated with restricted independent-prescribing status. Because practitioners across all regions already agree that validity should be the primary criterion for test selection (Section 3.6), interventions to increase uptake of evidence-based advanced diagnostics are more likely to succeed by addressing cost and workflow burden than by further validity evidence. Training programs should ensure low-cost, well-validated tools such as standardised symptom questionnaires remain consistently taught, given their current under-endorsement in SA despite requiring no specialist equipment. Finally, the tiered diagnostic framework proposed here (Figure 8) offers a starting point for structuring minimum-standard care pathways in resource-constrained settings, though formal validation against patient outcomes is required before adoption.
